# Loneliness and Well-Being during the COVID-19 Pandemic: The Moderating Roles of Personal, Social and Organizational Resources on Perceived Stress and Exhaustion among Finnish University Employees

**DOI:** 10.3390/ijerph18137146

**Published:** 2021-07-03

**Authors:** Jaana-Piia Mäkiniemi, Atte Oksanen, Anne Mäkikangas

**Affiliations:** Work Research Centre, Faculty of Social Sciences, Tampere University, 33014 Tampere, Finland; atte.oksanen@tuni.fi (A.O.); anne.makikangas@tuni.fi (A.M.)

**Keywords:** loneliness, COVID-19, personal demands, stress, exhaustion, personal resilience, social belonging, organizational support

## Abstract

The aim of this study is to investigate whether personal, social and organizational level resources can buffer against the negative effects of perceived loneliness on stress and exhaustion. The data was collected from Finnish university employees (*n* = 1463) in autumn 2020 via an electronic survey. Of the respondents, about 78% were working remotely, and 64% were female. Hierarchical multiple regression analyses were used to analyze the main and moderating (i.e., buffering) effects. The results indicated that perceived loneliness was directly and positively associated with stress and exhaustion. Further, as hypothesized, personal resilience moderated the relationship between loneliness and stress and exhaustion, and organizational support moderated the relationship between loneliness and stress. Unexpectedly, organizational support did not moderate the loneliness–exhaustion relationship. Moreover, a sense of social belonging was not associated with stress and exhaustion, nor did it moderate loneliness and well-being relationships. The results demonstrate the importance of personal resilience and organizational support in enhancing well-being in organizations during the COVID-19 pandemic. Future research directions and practical ways to promote resilience and to increase organizational support are discussed.

## 1. Introduction

The WHO declared the COVID-19 outbreak a pandemic on 11 March 2020. Since then, the pandemic has dramatically affected the social lives of the world population. In most societies, number of human contacts has been strictly limited by restrictions imposed by authorities, reducing the frequency of social contacts and of face-to-face social interactions at work and leisure [1]. Such a massive scale of social distancing due to the COVID-19 pandemic may have impacted the basic human need for social belonging and increased the likelihood of loneliness [2,3,4]. For example, during COVID-19, about 36% of British respondents felt lonely sometimes or often [5], and in the US, loneliness increased significantly from April to September 2020 [6]. Further, Finns in general, and especially young adults in Finland, experienced more loneliness than other Europeans during COVID-19 [7].

Loneliness can be defined as the distressing subjective feeling of lacking a social network or companion, and refers to a perception that one’s social relationships are inadequate in light of one’s preferences [8]. Hence, being by definition a subjective perception or feeling, even people with frequent social contacts may feel lonely, and on the other hand, those with few social contacts may not feel lonely [9]. Loneliness has been shown to be linked to many negative outcomes, such as higher risks for mental health problems, morbidity and all-cause mortality [10,11,12,13]. Although loneliness was a significant concern even before the pandemic, it is being more widely recognized due to the pandemic [4,14]. Therefore, personal and workplace factors which can protect against the negative effects of perceived loneliness on well-being need to be identified, and this is the aim of the present study.

### 1.1. Loneliness as an Interpersonal Stressor and a Personal Demand

Loneliness can be seen as an interpersonal stressor giving rise, for instance, to negative emotions and negative physiological reactivity, which in turn is conducive to unhealthy activity of the nervous system [15]. In line with this, adults experiencing loneliness are also characterized by higher levels of anxiety, negative mood and stress [11]. Feeling lonely is also linked to greater morning rises in levels of cortisol, the primary stress hormone [16]. It is assumed that lonely and socially isolated individuals, due to their lack of social networks and support, may suffer more than others from stress since social networks have the potential to protect against the detrimental effects of stressors [15].

Findings during the COVID-19 pandemic support the view of the detrimental effects of loneliness. For instance, in autumn 2020, greater loneliness among Finnish workers was associated with higher COVID-19 anxiety [17]. Further, at the height of the social distancing restrictions, loneliness was associated with increased depression among Canadian respondents [14]. It is noteworthy that some people feel lonelier than others during the pandemic. For instance, risk factors for loneliness in spring 2020 among UK adults were younger age and being separated or divorced [18]. In addition, during the strict two-week-long social distancing phase in Norway, single people and those with psychiatric diagnoses experienced loneliness most often [19]. Among Canadian respondents, younger females, individuals with lower income and those living alone experienced greater loneliness [14].

We conceptualize loneliness as a personal demand with a potentially negative influence on job-related well-being. The construct of personal demands is one of the most recent additions to the Job Demands–Resources (JD-R) model [20,21]. Although personal demands have been studied with the theoretical framework of the JD-R, no established definition has been proposed [22]. According to Salmela-Aro and Upadyaya [23], personal demands are individual characteristics that are reflected in employees’ effort in their work. Examples of the personal demands studied include, among others, high performance expectations at work [21], having a long-term illness, taking care of aging parents, experiencing financial problems, having demanding duties in personal life and relationship problems and demands outside of work (e.g., break-up) [22,23,24]. In addition, during COVID-19, when Chinese remote workers were interviewed, they reported that one of their key challenges was loneliness [25].

Loneliness may affect work effort, performance and job-related well-being, because it is linked, for instance, to general stress levels, poorer overall cognitive performance, negative reactivity and mood. It is highly plausible that this kind of negativity may spill over to the work domain and make it difficult to work collaboratively, while poor cognitive performance may influence and impair the quality of work performance [26]. The empirical findings support the idea that non-work personal demands are also involved in the health impairment process, referring to a process in which high demands increase the risk for poor well-being and lead to negative outcomes, such as health complaints [21]. For instance, it has been shown that employees experiencing high relationship demands typically belonged to the group among whom burnout increased over time [24], and caregiving demands and financial problems have been associated with higher levels of job burnout [23]. In a recent longitudinal study, general subjective loneliness was predictive of future work disability, and depression partly mediated the longitudinal relationship between loneliness and work disability [27]. Based on the prior findings, our hypotheses were:

**Hypothesis** **1.** *Perceived loneliness is associated with higher perceived stress.*

**Hypothesis** **2.** *Perceived loneliness is associated with higher emotional exhaustion.*

### 1.2. Resilience, Social Belonging and Organizational Support as Potential Protective Factors

Various workplace resources have been shown to be directly associated with better job-related well-being [28]. According to the JD-R model, divergent job and personal resources can also buffer against the negative impact of job and personal demands on work well-being [20,21]. In the present study, we focus on the individual, group and organizational level resources, namely resilience, social belonging and organizational support. We suggest that they have potential to protect against the negative effects of perceived loneliness, conceptualized as a personal demand.

#### 1.2.1. Resilience as a Personal Resource

In the present study, resilience is defined as a person’s capacity to bounce back or recover quickly from a significant source of stress [29,30]. More concretely, individuals with high levels of resilience tend to bounce back quickly after experiencing a stressful situation. Moreover, it does not take them long to recover from a demanding event, and they usually come through difficult times with little trouble.

We conceptualize resilience as a personal resource that encourages adequate adaptation to significant stressors [29,30]. According to the JD-R model, employees use such personal resources to deal with personal and job demands [20,21,31]. Personal resources may be directly associated with better job-related well-being, but they may also buffer against the negative impacts of job and personal demands on work well-being [20,21]. Additionally, the conservation of resources model (COR) highlights the importance of personal resources in maintaining, protecting and promoting well-being [32].

In line with the assumptions of the JD-R model, personal resilience has been shown to be directly associated with reduced burnout symptoms, such as lower emotional exhaustion [33,34,35]. According to the latent profile analyses, Finnish employees who experienced high personal resources (including resilience) were more likely to belong to the high work engagement group than to the group among whom burnout increased over time [24]. In addition, personal resilience has been shown to be related to less perceived stress [30]. Moreover, in line with the JD-R model, evidence suggests that resilience can buffer against the negative impact of demands on work well-being [36,37]. For example, personal resilience was shown to reduce the negative impact of time pressure on emotional exhaustion [38]. During COVID-19, the importance of personal resilience may be even greater. For instance, among Australian health workers in spring 2020, high resilience was associated with less endorsement of anxiety and post-traumatic stress [39]. Further, COVID-19-related loneliness increased the likelihood of sleep problems, and this association was especially strong among those with lower resilience [40]. In light of these findings, we expected that:

**Hypothesis** **3.** *Personal resilience will be directly associated with lower stress and emotional exhaustion.*

**Hypothesis** **4.** *Personal resilience will moderate the relationship between loneliness and stress and exhaustion. That is, the relationship between loneliness and well-being is stronger among those employees with a lower (vs. higher) level of resilience.*

#### 1.2.2. Sense of Social Belonging as a Social Resource

Humans have a basic need to belong, form and maintain interpersonal relationships [2], and these relationships play an essential role in individuals’ well-being. For instance, social support has been shown to be directly related to improved well-being, and also to buffer against potentially adverse effects [41]. Accordingly, various social resources at work, such as feelings of social support, positive perception of team climate and good interpersonal relationships between employees, have consistently been shown to be associated with better employee well-being [28]. For instance, both work-related social support (e.g., co-worker and supervisor support) and non-work-related social support have been found to be negatively related to exhaustion [42,43,44]. However, the quality of social relationships matters: it seems that positive and balanced social relationships can provide social resources that are responsive to the needs caused by stressors and demands [45].

Feelings of social support and connection can be an important protective resource against the negative effects of loneliness during the pandemic [41]. As an example, in spring 2020, among UK adults, perceived social support was identified as a protective factor against loneliness [18]. Moreover, during a six-week nationwide lockdown in spring 2020, among a representative sample of Austrian citizens, greater social connectedness (i.e., greater size of social network) was associated with lower levels of perceived stress and fatigue [46]. In autumn 2020, those Finnish workers who reported higher social support also reported lower COVID-19 anxiety [17]. Overall, these results suggest that social resources can serve act as a protective factor in coping with the global pandemic. In the present study, we focus on social belonging, which is a sense of belonging to a certain group. The feeling of social belonging (e.g., how people feel that they belong in their institutions) is shown to be negatively associated with emotional exhaustion [47]. Based on these findings, we expected that:

**Hypothesis** **5.** *Social belonging will be directly associated with lower stress and emotional exhaustion.*

**Hypothesis** **6.** *Social belonging will moderate the relationship between loneliness and stress and exhaustion. That is, the relationship between loneliness will be stronger among those employees with lower (vs. higher) levels of social belonging.*

#### 1.2.3. Organizational Support as a Job Resource

According to the JD-R model and empirical findings, organizational support is a job resource that is associated with improved job-related well-being and can protect against the harmful effects of demands [20,21,28]. Various human resource management practices and activities can be interpreted by employees as indicative of organizational support and care, and the practices may assist work processes and enhance well-being [28,48,49,50].

During the COVID-19 pandemic, many organizations have implemented full-time remote work for their employees in response to the crisis. Consequently, more extensive remote work support was needed in organizations. The perceived organizational remote work task support is employees’ sense that their organization provides them with the necessary resources for remote work, such as information technology support, timely information, relevant work materials and decision-making authority [51,52]. The importance of different types of perceived organizational support has been shown during COVID-19 in various occupations. For instance, US restaurant frontline employees with higher levels of perceived organizational support during COVID-19 exhibited lower levels of emotional exhaustion [53]. Moreover, the perceived organizational support moderated the relationship between the extent of exposure with COVID-19 patients and job stress among Pakistani medical doctors [54].

Therefore, we expected that:

**Hypothesis** **7.** *Organizational support will be directly associated with lower stress and emotional exhaustion.*

**Hypothesis** **8.** *Organizational support will moderate the relationship between loneliness and stress and exhaustion. That is, the relationship between loneliness will be stronger among those employees with lower (vs. higher) levels of organizational support.*

### 1.3. Aim of the Study

Taken together, the aim of the present study is to explore whether personal, social and organizational level resources can protect against the negative effects of perceived loneliness on stress and exhaustion. The respondents are Finnish university employees who were advised to work remotely due to governmental recommendations. These recommendations came into effect at the beginning of March 2020 and will end in July 2021. At the time of data collection, study participants had been working remotely for about eight months.

This study contributes to the literature in different ways. First, we study protective factors against the potential negative effects of loneliness. We consider that this is important, since many studies have focused on the mean levels and antecedents of loneliness during COVID-19 [19], and therefore less is known about the protective factors. Knowledge about the protective factors also helps to develop strategies to tackle the detrimental effects of loneliness in organizations as remote and hybrid work is forecast to continue in the future. Second, we have adopted an integrative approach and study several potential protective factors simultaneously, and at three different levels, namely individual, social and organizational levels. The selected approach makes it possible to compare the effects of the factors and identify the most influential moderators. Third, the study is, to the best of our knowledge, the first to address loneliness as a personal demand from the perspective of the JD-R model. The findings of the study can thus inform the conceptualization of personal demands, as well as theoretical model development. Fourth, our sample consists of an occupational group that was compelled to engage in long-term remote work. This type of group has been only little studied during the COVID-19 pandemic since many studies on well-being during COVID-19 have concerned those working at the front line (e.g., healthcare) [39].

## 2. Materials and Methods

### 2.1. Participants and Procedures

The data used in this study were collected as a part of the research project “Safely remotely—occupational well-being and its management in telework”, funded by the Finnish Work Environment Fund. The overall aim of the research project was to examine university employees’ experiences of remote work during the COVID-19 pandemic. The data used in this study were collected from a multi-faculty university located in western Finland from 28 September to 11 October 2020. Cross-sectional data were collected using the LimeSurvey tool. The electronic survey was sent to the work email addresses of 3788 university employees via the university’s general mailing list. Participants were informed about the survey before it was sent to them, and one reminder was used. The study was approved by the Institutional Review Board of the University of Turku, date 18 May 2020. All subjects provided their informed consent to inclusion before they participated in the study. In total, 1487 participants completed the survey, resulting in a response rate of 39%.

The sample of the present study (*n* = 1463) comprises all those who had employment contracts with the university, i.e., grant holders supported by (personal) grants from external funding agencies were excluded from the data. Half of the respondents were academic staff (50%), comprising, for example, full professors, lecturers, (senior) researchers and academic assistants. Of the respondents, 38% belonged to the group of administrative and technical personnel. The rest of the respondents belonged either to the group of managers (6%) or doctoral students with employment contracts (6%). Of the sample, 64% were women (0.7% declined to report their gender and 4.1% preferred not to answer the question), the average age was 45 years (SD = 10.66), 76% had a partner and the majority had either a master’s (46.5%) or doctoral (33%) degree. The vast majority of the participants were working remotely during the study (78%). Compared to the personnel structure of the university in question, the participants in our study were somewhat older (45 years vs. 44 years), and women were slightly over-represented in our data (64% vs. 59%). In 2020, the total number of staff working in 13 Finnish universities administered by the Ministry of Education and Culture was 30,050 [55]. Compared to the general personnel structure of Finnish universities, women (64% vs. 59%), as well as support and administrative personnel (44% vs. 40%) (i.e., those working with other than academic duties), were slightly over-represented in our data [55].

### 2.2. Measures

Means, standard deviations and reliabilities of the scales used are presented in Table 1.

Loneliness was measured on the Short Loneliness Scale [8,56]. It contained three items (“How often do you feel lonely?”, “How often do you feel left out?”, “How often do you feel isolated from others?”) which were rated on a 3-point scale (1 = hardly ever, 3 = often). The scale was previously used to study loneliness during COVID-19 [17].

Personal resilience was measured with three items adapted from the Brief Resilience Scale [29], namely “I tend to bounce back quickly after hard times”, “It does not take me long to recover from a stressful event” and “I usually come through difficult times with little trouble”. A Finnish translation of the scale provided by Rantanen and colleagues [57] was used. The items were assessed on a 5-point Likert-type scale (1 = totally disagree, 5 = totally agree).

Sense of social belonging was measured by asking “How strongly do you feel you belong to a “family,” “friendship group” and “work community”?” on a 7-point scale (1 = not at all, 7 = very closely) [58,59]. The composite variable of the above-mentioned three items was used here, representing the overall experience of social belonging.

Organizational support was measured with six new items specifically developed by the research team for the purposes of this study: (1) “The top management of the university have communicated clearly about the current exceptional circumstances”, (2) “My practical questions have been answered quickly enough”, (3) I have received enough instructions on performing my tasks and duties from home”, (4) “I have received support for my work when I have encountered difficulties”, (5) “I have received enough instructions on using the electronic systems and tools (such as Teams, Zoom, Panopto, Moodle)” and (6) “The electronic systems and tools (such as Teams, Zoom, Panopto, Moodle) have worked well technically”. The items were scored on a 5-point rating scale (1 = strongly disagree, 5 = strongly agree).

The single-item measure of stress has been found to adequately capture experience of perceived stress [60]. A rating on a 5-point Likert-type scale (1 = not at all, 5 = very much) was provided in response to the question “Stress means a state in which a person feels tense, restless, nervous or anxious, or is unable to sleep at night because his/her mind is troubled all the time. Do you feel this kind of stress these days?”.

Exhaustion was measured with three items from the Burnout Assessment Tool [61], capturing feelings of fatigue that develop as one’s emotional energies become drained at work (e.g., “At work, I feel mentally exhausted”). The items were rated on a 5-point scale (1 = never, 5 = always).

Gender (1 = men, 2 = other than men), age (a continuous variable) and marital status (1 = in a relationship, 2 = no relationship) were used as covariates in the analyses, as they have been in earlier studies associated with loneliness and well-being [7,14].

### 2.3. Data Analysis

Hierarchical multiple linear regression analyses were used to examine the main effect of loneliness and the possible buffering (i.e., moderating) effects of resilience, social belonging and organizational support on loneliness–well-being (perceived stress and exhaustion) relationships. Both stress and exhaustion were regressed on the antecedent sets in six steps, as follows: (1) demographics (gender, age, marital status), (2) loneliness, (3) resilience, (4) social belonging, (5) organizational support, and (6) the interaction terms between loneliness and resiliency, social belonging and organizational support. The magnitude of R^2^ change at each step of the analysis was used to determine the variance explained by each antecedent or antecedents set. The standardized beta values reported were used to determine the effect of each variable on stress and exhaustion. Analyses were conducted using SPSS Statistics Version 27 (IBM, Armonk, NY, U.S.). Basic assumptions of regression analysis on normality and homoscedasticity of residuals were tested and the assumptions were met. Outliers were also checked, and there was no apparent multicollinearity.

## 3. Results

### 3.1. Descriptive Statistics

Correlations of all the study variables are presented in Table 1. As shown, loneliness correlated positively both with perceived stress (r = 0.42, *p* < 0.001) and exhaustion (r = 0.43, *p* < 0.001). Moreover, resilience, social belonging and organizational support all correlated negatively and statistically significantly with perceived stress and exhaustion. Personal, social and organizational resources also correlated with each other (r = 0.25–0.30, *p* < 0.001), as also did perceived stress and exhaustion (r = 0.67, *p* < 0.001). Of the background variables, women (and those not identifying with female or male gender or who declined to report their gender) (r = 0.08–0.09, *p* < 0.01) and younger employees (r = −0.20–−0.18, *p* < 0.001) perceived more stress and exhaustion than the others. There was no difference in levels of loneliness between genders (*p* > 0.05), but single individuals (M = 1.83, SD = 0.65) reported higher levels of loneliness than those in relationships (M = 1.60, SD = 0.58, F (1) = 24.77, *p* < 0.001).

### 3.2. Results of Regression Analyses

The results of the multiple regression analyses are presented in Table 2. After controlling for the demographics at the first step, entering loneliness at step 2 lent support to the hypothesized main effects. Loneliness explained a substantial proportion of the variance in both perceived stress (15%) and exhaustion (16%). That is, the higher the level of loneliness, the more stress (β = 0.27, *p* < 0.001) and exhaustion (β = 0.28, *p* < 0.001) were reported. Consequently, our Hypotheses 1 and 2 were supported. The significant main effects were also evident for resilience (β = −0.33 and β = −0.38, *p* < 0.001) and organizational support (β = −0.12 and β = −0.11, *p* < 0.001) on both stress and exhaustion, respectively (see Table 2). Hypotheses and 7 were thus fully supported. Hence, the higher the reported level of resilience and support provided by the organization, the less stress and exhaustion were reported. These variables also moderated the loneliness–well-being relationships. According to our Hypothesis 4, resilience moderated the effects of loneliness on both stress (β = 0.08, *p* < 0.01) and exhaustion (β = 0.11, *p* < 0.001), and the interaction terms significantly contributed to the explained variance.

**Figure 1:**
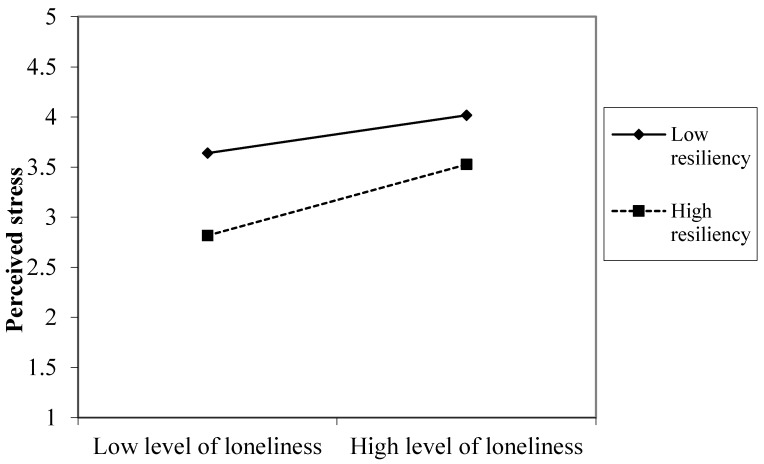
A significant interaction effect between loneliness and resilience on perceived stress.

**Figure 2:**
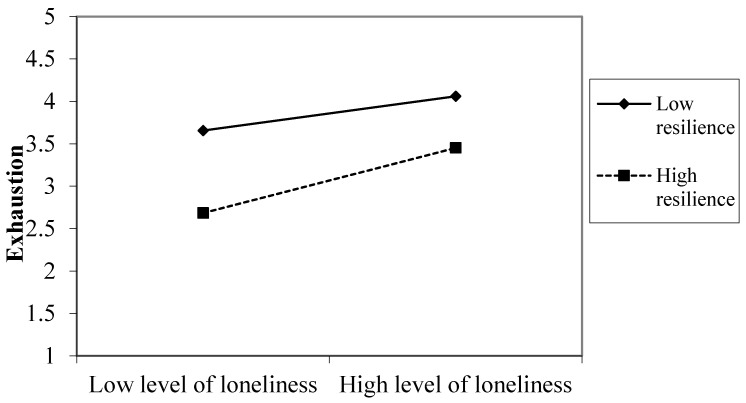
A significant interaction effect between loneliness and resilience on exhaustion.

Graphical representations of the significant two-way interactions (see Figure 1 and Figure 2) were made using the standardized regression coefficients of the regression lines for employees high (1 SD above the mean) and low (1 SD below the mean) on resilience. Figure 1 shows that employees with high levels of resilience suffered less from perceived stress under high levels of loneliness compared to employees with low levels of resilience. However, the beneficial effect of resilience on perceived stress was even more marked in a situation of low levels of loneliness. A similar moderator effect was evident for exhaustion, as shown in Figure 2: employees with low levels of resilience reported more exhaustion under high levels of loneliness compared to employees with high levels of resilience. However, it should be noted that resilient employees suffered less from exhaustion, especially in conditions of low loneliness.

**Figure 3:**
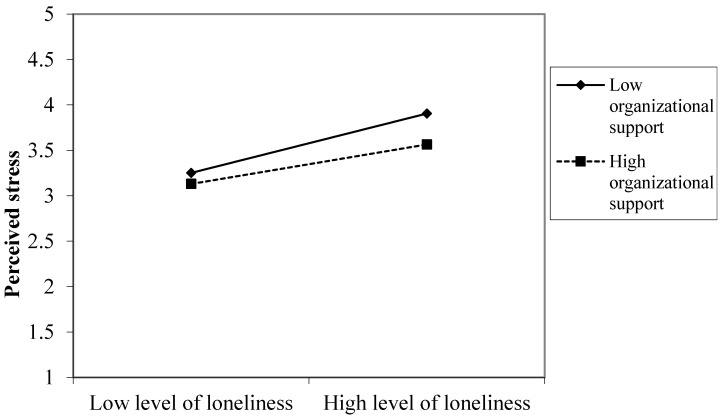
A significant interaction effect between loneliness and organizational support on perceived stress.

Organizational support, moreover, moderated the loneliness–stress relationship, thus lending partial support to our Hypothesis 8 (see Table 2). Figure 3 shows that the relation between loneliness and perceived stress was more marked for employees experiencing low organizational support. Thus, employees experiencing low organizational support suffered more from perceived stress under high levels of loneliness than did employees reporting high organizational support. However, when loneliness was at a low level, the level of perceived stress was the same for high and low levels of organizational support.

Social belonging did not contribute to explaining the variance of perceived stress or exhaustion. Additionally, the interaction terms between loneliness and social belonging turned out to be nonsignificant (see Table 2). Therefore, our Hypotheses 5 and 6 were not supported. Altogether, the models explained 32% and 37% of the variance of the perceived stress and exhaustion, respectively.

## 4. Discussion

The main aim of this study was to investigate whether among Finnish university employees, personal resilience, social belonging and organizational support buffered against the potentially negative effects of perceived loneliness on stress and exhaustion. This study is among the first to study loneliness as a personal demand from the perspective of the JD-R model [21]. It also enhances our understanding of the relationships between loneliness, workplace resources and well-being during the pandemic.

First of all, as hypothesized, perceived loneliness was associated with stress (H1) and exhaustion (H2), where the higher the level of loneliness, the more stress and exhaustion were experienced. Taken together, these results support the existing evidence on the detrimental influence of loneliness on well-being in general [10,11,13] and during COVID-19 in particular [17]. Personal resilience was directly and negatively associated with stress and exhaustion: the higher the level of personal resilience, the less stress and exhaustion were experienced. Personal resilience also moderated the effect of loneliness on stress and exhaustion, thereby supporting the Hypotheses 3 and 4. However, interpretation of the interaction showed that the beneficial effects of resilience on perceived stress and exhaustion were more marked in a situation of low levels of loneliness than in a situation of high loneliness. There may be several explanations for these effects. For example, in the present study, personal resilience was conceptualized and measured as person’s capacity to ‘bounce back’ or recover quickly from a source of stress. As loneliness in the COVID-19 situation represents a chronic and enduring stressor, it may be that personal resources are not enough to cope with it. Moreover, resiliency represents personal resources that may not suffice to cope with severe loneliness, but instead also require social resources in order to facilitate well-being.

Overall, the results support the basic assumptions of the JD-R model [20,21], namely that personal resources are associated with improved well-being, and that they can also protect against demands. During the pandemic, the relevance of personal resilience may increase because of the constant need to cope with novel and changing stressors, such as to being quarantined or fear of contracting coronavirus, together with novel job demands (e.g., requirement for remote work or risks associated with working at the front line). Additionally, recommendations, restrictions and actions are constantly evaluated and changed due to the prevailing pandemic situation. Consequently, there is a need to recover quickly from stressful changes. Personal resilience has been only little studied as an individual resource associated with work-related well-being [28]. However, according to our results, it is important for individual resources to be integrated in future studies. Our results also extend the understanding and conceptualization of personal demands and suggest that loneliness can also be treated as a personal demand [21,23,24].

Our findings, moreover, indicated that perceived organizational support was directly and negatively associated with exhaustion and stress: the higher the level of perceived organizational support, the less stress and exhaustion were experienced. Organizational support also moderated the loneliness–stress relationship, as hypothesized, but did not moderate the loneliness–exhaustion relationship. Thus, Hypothesis 7 was fully, and Hypothesis 8 partially supported. These findings concur with earlier findings on the importance of organizational support for work well-being in general [20,21,28,50], and during the pandemic in particular [53,54]. Organizational support was measured in the present study on a scale focusing especially on organizational support during COVID-19 with reference to remote work. As an example, communications on restrictions and instructions for using electronic systems and tools when working remotely due to the pandemic were highlighted. Although the results are not entirely comparable to those of earlier studies, we suggest that in the future, the features of the prevailing situation (e.g., “forced” remote work due to the pandemic) should be considered when selecting the scales to measure organizational support, since different types of support are needed in different situations. On the other hand, we assume that one reason for organizational support not moderating loneliness–exhaustion relationships may be due to our focus on organizational support for remote work. Such support might well alleviate acute stress, for instance when a problem associated with the use of electronic systems and tools is solved, but such support is not sufficient in cases of more chronic exhaustion.

Contrary to expectations, social belonging was associated with neither stress nor exhaustion, and did not moderate the relationships between loneliness and well-being. Hypotheses 5 and 6 were therefore not supported by the results. Since social belonging was not associated with well-being, we propose that in the future, other constructs, such as emotional and instrumental social support (e.g., collegial and supervisory), could be included in the analyses. Additionally, quality of social support or social interaction should be considered since quality of social interaction has been shown to be relevant for well-being [43,45,62]. Moreover, although the respondents felt quite closely connected to their families, friends and the work community, this sense of social belonging was not able to buffer against the negative effects of loneliness. Those perceiving a close connection and sense of belonging may also suffer more from social isolation since they feel connected with people but are denied face-to-face interaction with them.

### Limitations

The main limitation of this study concerns the fact that the study design was cross-sectional, making it impossible to draw conclusions about the direction of causality, for example, whether loneliness causes stress and exhaustion, or whether stress and exhaustion cause more loneliness to be experienced. Further, it is likely that feelings and experiences have changed and developed during the pandemic due to changing risk levels and restrictions, for example [6]. We therefore utilized a sample of participants whose remote work had already lasted eight months. In future studies, it would be beneficial to analyze the direction of causality and developmental trajectories using longitudinal study designs. However, cross-sectional study design also has its value: with one time point, one can provide a detailed picture of experiences related to the exact time period, and the design suits well for investigating moderators, which was the main aim of the study. Moreover, our data collection was based on self-evaluation, thus the possibility of common method variance bias cannot be totally ruled out. However, the response scales used varied between variables, which may have reduced the problem of common method bias associated with self-report methods [63]. In addition, the short scale for general social belonging might have afforded superior reliability, but on the other hand, it is efficient in capturing a wide range of connectedness from various sources. The use of only one sample naturally decreases the generalizability of the findings. Therefore, it is still important for future research to rely on more diversified samples in terms of professions.

The respondents in the present study were Finnish university employees surveyed at the turn of September and October 2020. At the time of data collection, most of them had been working remotely for about eight months. We suggest that our findings are likely to be generalizable not only to those working in universities but also to those knowledge workers who work remotely. However, it is worth noting that Finland is a Nordic welfare society, providing reasonable social security benefits and public services, which may be reflected in general stress and exhaustion levels. Further, during COVID-19 in Finland, the number of those working remotely was very high [64], and most of the participants of the current study were also working remotely. A fast and extensive shift to remote work has been possible and likely less stressful than in some other countries due to the fact that Finland is a highly digitalized country and many workers were already used to working with digital technologies [65]. Moreover, the Finnish higher education and university systems have their own special characteristics [66], which may be reflected in participants’ experiences. When the well-being of academics representing nineteen different higher education systems and countries was studied, academics from Finland, Japan, Canada, The Netherlands and Korea formed a group that was highly satisfied and highly stressed at the same time [67]. The finding was explained by the notion that the higher education systems of the above-mentioned countries provide good working conditions, but on the other hand, they have aggressively adopted performance-based management systems. Finally, it is important to take into account when interpreting the results that the number of confirmed COVID-19 patients has been low in Finland (population about 5.5 million) in comparison with European and adjacent countries. For instance, from 3 January 2020 to 17 June 2021, there have been 93,923 confirmed cases and 964 deaths in Finland reported to WHO [68]. That is about 1670 cases and 17 deaths per 100,000 population. In the corresponding period in Sweden, there were per 100,000 population a total of 10,502 confirmed cases and 141 deaths, and in Estonia, 9832 cases and 95 deaths per 100,000 population. The reasons for the relatively low number of cases and deaths in Finland may relate to the fact that the pandemic spread quite late to Finland, enabling early implementation of national and organizational restrictions and recommendations, and the health system managed to care for the patients with good results [1,69].

## 5. Conclusions

Our main findings demonstrated the importance of two resources—personal resilience and organizational support—in enhancing well-being during the COVID-19 pandemic. Based on these, we make two practical recommendations for organizations. First, in terms of enhancing personal resilience, one option could be to intentionally promote the ability to focus on positive experiences and to intensify and prolong positive feelings [70]. In practice, colleagues and supervisors could help each other to focus more on positive aspects, for instance by giving more positive feedback and noticing and remembering positive moments and mentioning them more frequently at work [71,72]). Second, in terms of organizational support, our findings indicated that it is especially important to invest effort in clear and up-to-date communication, creating new instructions on how to work in a novel situation in general and related to digital technologies in particular [51,52]. Moreover, it is important to provide special support (e.g., targeted occupational healthcare services, individual working arrangements) when employees face difficulties. Finally, when working remotely with digital technologies, it is crucial to ensure that employees have the ability to use them and that the technologies are usable and reliable.

## Figures and Tables

**Table 1:** Descriptive information on the study variables (*n* = 1337–1443).

Variables	M/%	SD	α	1	2	3	4	5	6	7	8
(1) Gender ^a^	31.20 ^d^	0.47	-								
(2) Age	45.09	10.66	-	−0.02							
(3) Marital status ^b^	75.8 ^e^	0.38	-	−0.05	−0.07 *						
(4) Loneliness	1.64	0.60	0.80	0.02	−0.19 ***	0.14 ***					
(5) Resilience	3.38	0.82	0.82	−0.10 ***	0.09 **	−0.04	−0.33 ***				
(6) Social belonging	5.29	1.01	0.61	0.10 ***	0.13 ***	−0.23 ***	−0.48 ***	0.30 ***			
(7) Org. support	3.84	0.76	0.85	−0.05	0.01	−0.02	−0.28 ***	0.27 ***	0.25 ***		
(8) Perceived stress ^c^	2.83	1.18	-	0.08 **	−0.20 ***	0.02	0.42 ***	−0.44 ***	−0.25 ***	−0.26 ***	
(9) Exhaustion	2.79	0.87	0.88	0.09 **	−0.18 ***	0.02	0.43 ***	−0.51 ***	−0.24 ***	−0.28 ***	0.67 ***

Note. ^a^ Gender: 1 = men, 2 = other than men. ^b^ Marital status: 1 = in a relationship, 2 = no relationship. ^c^ Perceived stress measured with a single question. ^d^ = percentage of men among participants, ^e^ = percentage of participants in a relationship. * *p* < 0.05, ** *p* < 0.01, *** *p* < 0.001.

**Table 2:** Results of multiple regression analyses with stress and exhaustion as dependent variables.

Perceived Stress						Exhaustion				
Variables	B	SE B	β	ΔR^2^	R^2^	B	SE B	β	ΔR^2^	R^2^
Step 1: Demographics				0.043 ***	0.043 ***				0.046 ***	0.046 ***
Gender ^a^	0.06	0.06	0.02			0.07	0.04	0.04		
Age	−0.01	0.00	−0.13 ***			−0.01	0.00	−0.09 ***		
Marital status ^b^	−0.13	0.08	−0.04			−0.07	0.06	−0.03		
Step 2: Loneliness	0.53	0.06	0.27 ***	0.151 ***	0.194 ***	0.42	0.04	0.29 ***	0.164 ***	0.210 ***
Step 3: Resilience	−0.46	0.04	−0.33 ***	0.105 ***	0.299 ***	−0.39	0.03	−0.38 ***	0.134 ***	0.344 ***
Step 4: Social belonging	0.04	0.03	0.03	0.000	0.299 ***	0.02	0.02	0.02	0.000	0.344 ***
Step 5: Org. support	−0.18	0.04	−0.12 ***	0.012 ***	0.311 ***	−0.13	0.03	−0.11 ***	0.011 ***	0.355 ***
Step 6: Interaction terms				0.007 **	0.318 ***				0.010 ***	0.365 ***
Loneliness * resilience	0.09	0.03	0.08 **			0.09	0.02	0.11 ***		
Loneliness * s. belonging	−0.15	0.03	−0.02			0.00	0.02	0.00		
Loneliness * org. support	−0.05	0.03	−0.05 *			−0.01	0.02	−0.01		

Note. ^a^ Gender: 1 = men, 2 = other than men. ^b^ Marital status: 1 = in relationship, 2 = no relationship. B = unstandardized beta-coefficient from the final step, SE B = standard error of the unstandardized beta-coefficient, β = standardized beta-coefficient from the final step, ΔR^2^ = change in explanation rate in each step, R^2^ = explanation rate. * *p* < 0.05, ** *p* < 0.01, *** *p* < 0.00.

## Data Availability

The data is available from Anne Mäkikangas upon reasonable request. Data will be made publicly available in the Finnish Social Science Data Archive in 2025.
